# Protective Effects of Fucoidan Isolated from Celluclast-Assisted Extract of *Undaria pinnatifida* Sporophylls against AAPH-Induced Oxidative Stress In Vitro and In Vivo Zebrafish Model

**DOI:** 10.3390/molecules25102361

**Published:** 2020-05-19

**Authors:** Jae-Young Oh, Eun-A Kim, Sang In Kang, Hye-Won Yang, Bomi Ryu, Lei Wang, Jung-Suck Lee, You-Jin Jeon

**Affiliations:** 1 Department of Marine Life Science, Jeju National University, Jeju City 63243, Korea; ojy0724@naver.com (J.-Y.O.); koty221@naver.com (H.-W.Y.); ryu.bomi@gmail.com (B.R.); 2 Jeju International Marine Science Center for Research & Education, Korea Institute of Ocean Science and Technology, Jeju City 63349, Korea; euna0718@kiost.ac.kr; 3 Department of Seafood and Aquaculture Science, Gyeongsang National University, Tongyeong 53064, Korea; progment@naver.com; 4 Marine Science Institute, Jeju National University, Jeju City 63333, Korea; comeonleiwang@163.com; 5 Research Center for Industrial Development of Seafood, Gyeongsang National University, Tongyeong 53064, Korea

**Keywords:** zebrafish, antioxidant, *Undaria pinnatifida* sporophylls, enzyme-assisted extraction, fucoidan

## Abstract

Fucoidan is a fucose-enriched polysaccharide, obtained from brown algae, with demonstrated antioxidant properties. However, traditional extraction methods using water or chemical-based extraction methods have reduced yield and produced hazardous by-products. In this study, we isolated fucoidan at a high yield using enzyme-assisted extraction; the Celluclast enzyme assisted extract of *Undaria pinnatifida* sporophylls (FCUS). To examine the antioxidant properties of FCUS, oxidative stress was induced with 2,2′-azobis (2-methylpropionamidine) dihydrochloride (AAPH) in Vero cells and zebrafish model. FCUS was composed of 30.4% sulfate and 52.3% fucose. Pre-treatment of Vero cells with FCUS dose dependently inhibited AAPH-induced reactive oxygen species (ROS) production. Moreover, FCUS remarkably reduced cell death, ROS generation, and lipid peroxidation production in zebrafish larvae. Overall, these findings indicate that the sulfate-rich fucoidan of FCUS, obtained with an eco-friendly process, could be implemented as a beneficial antioxidant agent in the functional food industry.

## 1. Introduction

Zebrafish (*Danio rerio*) are vertebrates that have special advantages over other experimental animal models, due to their smaller size, high breeding capacity, and short life cycle [1]. The zebrafish model has already been explored for drug screening and toxicology involving a variety of transgenic and wild type models due to its specificity.

Zebrafish larvae are transparent in nature. This facilitates the examination of different body parts and organs, such as bones, blood vessels, and different tissues like nerves. Hence, zebrafish is a handy and practical in vivo model for a variety of applications, including functional studies of food ingredients [2].

Generally, seaweeds contain about 90% water, and the other components include carbohydrates, proteins, and trace amounts of polyphenols and sterols. Raw materials (prior to extraction) contain fewer quantities of functional components per unit weight; therefore, for product development, extraction and purification of these raw materials are required to increase their efficacy. Enzymatic hydrolysis or enzyme-assisted extraction methods can destroy the cell membrane [3], facilitating the isolation of functional components at high yields. Celluclast is an enzyme obtained from *Trichoderma resei,* and it efficiently hydrolyzes cellulose to glucose. In general, Celluclast is used to break down cellulosic material for the production of fermentable sugars. Simultaneously, this results in the reduction of viscosity and increased extraction yield of plant materials [4,5,6].

Fucoidan is a fucose-containing sulfated polysaccharide in brown algae (*Phaeophyceae*), comprising l-fucose as the main sugar, along with galactose, glucose, mannose, and sulfate ester groups [5,7,8,9]. Previous studies demonstrated that fucoidan possesses a wide range of biological activities, such as anti-tumor, antioxidant, anti-diabetes, anti-inflammatory, and immunomodulatory effects [2,10,11,12,13]. Fucoidan is typically extracted using hot water and acidic water using HCl as a solvent [6,14]. However, the extraction process involving water and organic solvents is time-consuming and results in the release of some toxic compounds to the environment [15,16]. Therefore, the present study aims to develop a more environment friendly extraction method of fucoidan without releasing toxins while achieving a high yield and good quality of sulfate and fucose contents.

Enzyme-assisted extraction methods emerged as more eco-friendly extraction technologies, with relatively lower energy consumption and high yields of materials, such as polysaccharides, proteins, oils, and natural pigments [15,17]. Therefore, these methods were applied for the safe extraction and isolation of bioactive metabolites from seaweeds for food applications [15].

*Undaria pinnatifida* sporophylls, known as “Miyeokgwi” in Korea, have traditionally been considered as derelict by-products of *Undaria pinnatifida* leaves that are generally dumped at sea. However, many studies have reported various bioactivities of fucoidan isolated from *U. pinnatifida*, including anti-tumor, immune-stimulatory, anti-viral, and anti-coagulant activities [18]. Therefore, the value of fucoidan derived from *U. pinnatifida* is increasingly being recognized as a food and functional ingredient.

To improve its production, in the present study, we isolated fucoidan from *U. pinnatifida* sporophylls using an enzyme-assisted extraction method with Celluclast and measured its antioxidant activity using Vero cells and zebrafish larvae as in vitro and in vivo models, respectively, of oxidative stress induced by 2,2′-azobis (2-methylpropionamidine) dihydrochloride (AAPH).

## 2. Results and Discussion

### 2.1. Proximate Composition of the FCUS

The composition of FCUS is summarized in Table 1. FCUS contained 66.8% carbohydrates and 2.8% protein of the total dry weight. The monosaccharide composition analysis showed that fucose (52.3%) and galactose (44.5%) are the major sugars in FCUS. Besides, FCUS contained 30.4% sulfate. Therefore, the fucoidan content of FCUS was approximately 97% of the total dry carbohydrate content. Fucoidan is a sulfated polysaccharide derived from brown seaweed. It was enriched in l-fucose and sulfate ester groups and possesses demonstrated antioxidant activity [19]. To overcome the limitations and disadvantages of the conventional extraction of fucoidan using hot water, organic solvents, or acid solvents, we demonstrated the feasibility of adopted of enzymatic hydrolysis with Celluclast enzymes to extract fucoidan from sporophylls of the brown algae, *U. pinnatifida*.

The use of an enzyme-assisted extraction method has a substantial advantage for achieving high extraction yields for compounds, along with being an eco-friendly process with higher recovery, reduced solvent usage, and lower energy consumption, compared to non-enzymatic extraction methods [15]. Therefore, enzyme-assisted extraction methods have been widely used to obtain functional materials, such as oils, peptides, polysaccharides, and pigments [20,21,22,23]. Commercial fucoidan prepared from *Fucus vesiculosus* is mainly composed of fucose (44.1%) and sulfate (26.3%) [24]. Fucoidan previously isolated from Miyeokgwi, using HCl as the solvent, was obtained with a yield of only 4.9%, comprising of 28.9% sulfate and 50.9% or 53% fucose [25]. In this study, however, FCUS accounted for approximately 97.2% fucoidan content in the carbohydrate content, 6.2% yield, comprising 30.4% sulfate and 52.3% fucose, demonstrating improvement over both the commercial fucoidan and HCl extract. Accordingly, hydrolyzed *U. pinnatifida* sporophylls using Celluclast enzyme-assisted extraction is a safe and eco-friendly extraction method, resulting in high contents of sulfate and fucose.

**Figure 1:**
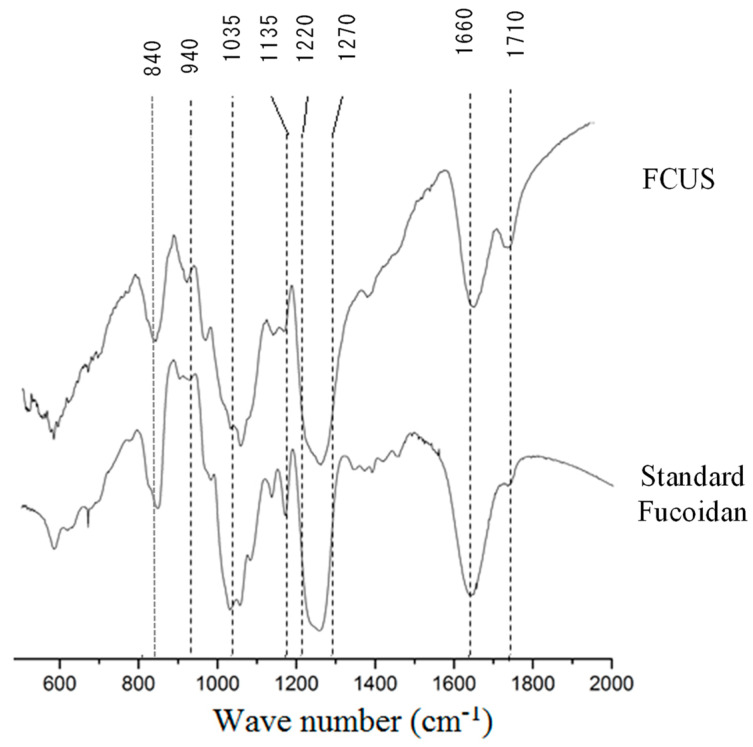
FT-IR spectra of FCUS and standard commercial fucoidan. FCUS and standard fucoidan were scanned between 600–2000 cm^−1^.

The FT-IR spectrum was recorded in the range of 600–2000 cm^−1^ to analyze the structural features of FCUS, such as glycosidic bonds and functional groups, which were compared to those of the commercial fucoidan (Figure 1). An intense peak centering at 840 and 1035 cm^−1^ indicated sulfation on C4 galactose and the stretching vibration of the glycoside bridge (C–O–C) [26]. The bands at 1135 and 1160 cm^−1^ represent stretching vibrations of the glycosidic C–O group and the aliphatic C=O bond, respectively [27]. The bands around 1220–1270 cm^−1^ suggest sulfate groups (O=S=O stretching), and that at 1710 cm^−1^ indicated a stretching vibration of the carbonyl groups in the carboxylic acid groups (C–O) of fucoidan [27]. FCUS showed a strong band at five peaks (840, 1035, 1220–1270, 1160, and 1710 cm^−1^), demonstrating a similar pattern to that of the commercial fucoidan.

### 2.2. Effect of FCUS on Intracellular ROS Generation in AAPH-Stimulated Vero Cells

Oxidative stress is associated with aging and inflammation, as well as several human diseases, such as Parkinson’s disease, rheumatoid arthritis, neurological disorder, and Alzheimer’s disease [28,29,30]. Commonly, ROS produced as a result of normal metabolism is eliminated by the endogenous cellular antioxidant system. However, elevated and abnormal levels of ROS have detrimental effects. External antioxidants would help to protect against cellular and tissue damage induced by excessive ROS production. Recently, natural antioxidants have been developed, and we have explored various natural antioxidants from marine organisms, especially algae [31,32]. External stimuli, such as drugs, radiation, and dust, could induce abnormal ROS production [33]. Therefore, remarkable ROS scavenging activity and low and non-toxic substances may be the ideal features for protection against and treatment of oxidative stress-related human diseases. According to previous studies, sulfated polysaccharides derived from seaweeds have potent antioxidant activity [34,35]. As noted above, FCUS contained high amounts of sulfated polysaccharides (66.8 ± 0.1%) and antioxidative monosaccharides, such as fucose and galactose.

**Figure 2:**
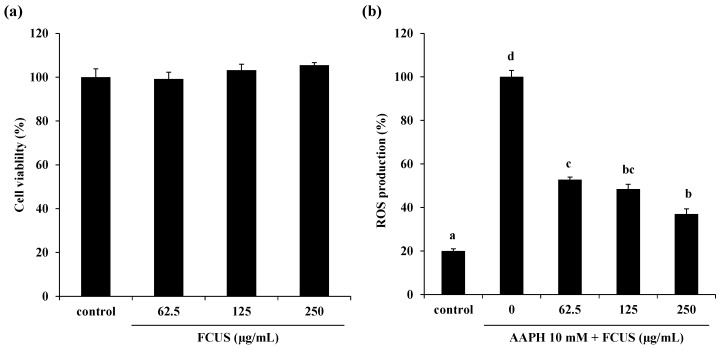
Cytotoxicity (**a**) and intracellular reactive oxygen species (ROS) scavenging activity (**b**) of FCUS in Vero cells. Each value is expressed as the mean ± SE. ^a–d^ Means not sharing a common letter are significantly different between groups (*p <* 0.05).

The MTT assay showed that FCUS did not have any cytotoxicity at the tested concentrations (62.5–250 µg/mL) compared with the control (Figure 2a). Therefore, further experiments were carried out using FCUS at concentrations lower than 250 µg/mL. We next measured the peroxyl radical scavenging activity to evaluate the intracellular ROS scavenging effects of FCUS against AAPH-induced oxidative stress in Vero cells. AAPH, a water-soluble azo compound, is commonly referred to as a free radical inducer and has been used in many studies on lipid peroxidation and characterization of antioxidants. As shown in Figure 2b, the ROS production was remarkably increased in the AAPH-treated group. However, the intracellular ROS production of cells treated with FCUS at 62.5, 125, and 250 µg/mL decreased in a dose-dependent manner (52%, 48%, and 36%, respectively).

### 2.3. Toxicity of FCUS in Zebrafish and the Effect of FCUS on ROS and Lipid Peroxidation Production in Zebrafish

Zebrafish is a valuable in vivo model for various oxidative stress assays and is used in studies of oxidative stress or protection against oxidative stress [36]. Oxidative stress, induced by AAPH and H_2_O_2_ could lead to elevation of heartbeat rate, ROS levels, lipid peroxidation, and cell death, as well as decrease the rate of survival, in zebrafish [36,37,38]. A previous study confirmed the antioxidant effects of fucoidan derived from *Ecklonia cava* (containing 51.8% carbohydrate, 20.1% sulfate, and 61.1% fucose) against AAPH-induced oxidative stress in the zebrafish model. It also improved survival rate and decreased heartbeat rate, production of ROS and lipid peroxidation, and cell death [13,39]. Consequently, AAPH-treated zebrafish were used as the model to determine the antioxidant effect of FCUS.

**Figure 3:**
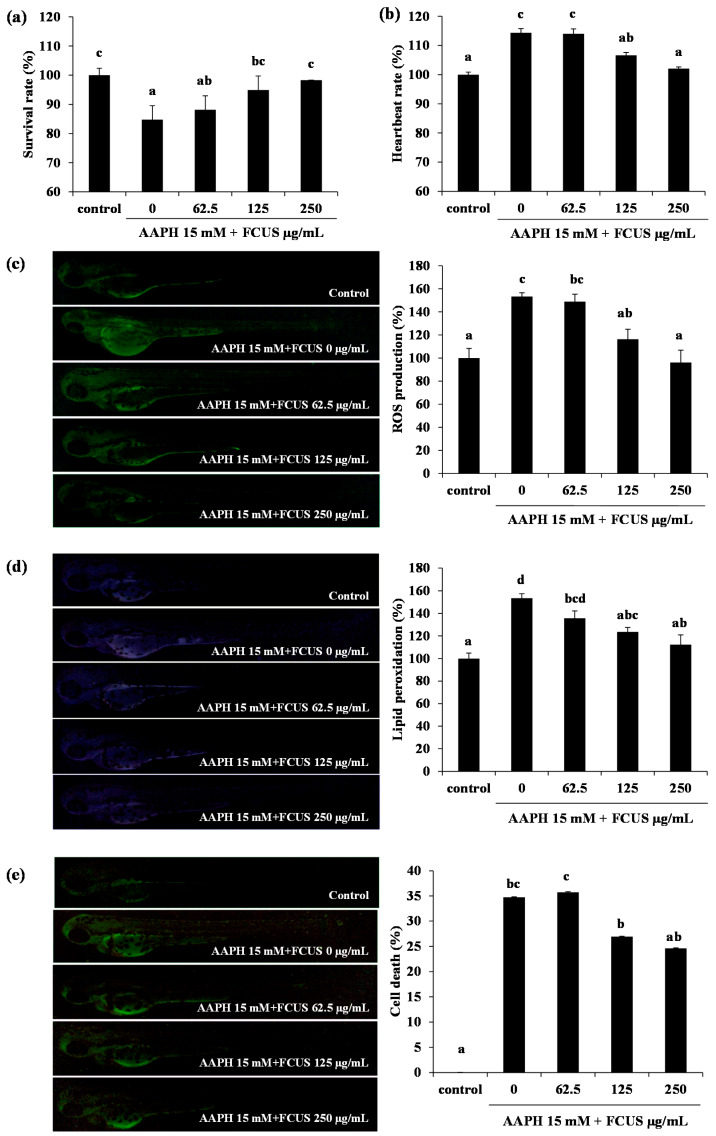
Effects of FCUS on the survival rate (**a**), heartbeat rate (**b**), reactive oxygen species (ROS) production (**c**), lipid peroxidation production (**d**), and cell death (**e**) in AAPH-treated zebrafish. Production of ROS, lipid peroxidation, and cell death were measured by fluorescence microscopy and analyzed using ImageJ software. Each value is expressed as the mean ± SE. ^a–d^ Means not sharing a common letter are significantly different between groups (*p <* 0.05).

The survival rate of zebrafish embryos was reduced to 84% in the AAPH-treated group, compared with that of the control group. However, the FCUS-treated groups (62.5–250 µg/mL) showed a significant increase in survival rates compared to the non-treated group, to 88%, 94%, and 95%, respectively (Figure 3a). The heartbeat rate of the AAPH-treated group increased to 114% compared with that of control group, and was significantly reduced with pre-treatment of FCUS at the concentrations of 62.5–250 µg/mL (Figure 3b).

Exposure of zebrafish larvae to AAPH resulted in a 150% increase in the ROS level, but it was decreased to 5%, 37%, and 57% with FCUS treatments at 62.5, 125, and 250 µg/mL, respectively (Figure 3c). Similarly, the AAPH-treated group showed a 150% increase in lipid peroxidation determined by the fluorescence of DPPP. However, the treatments with FCUS (62.5, 125, and 250 µg/mL) significantly suppressed the lipid peroxidation production and cell death in a dose-dependent manner (Figure 3d). Moreover, AAPH treatment significantly increased the cell death level to 33% compared with that of the control group, but it was decreased in a dose-dependent manner by FCUS treatments to 34%, 28%, and 23% at 62.5, 125, and 250 µg/mL, respectively (Figure 3e). AAPH is well known to strongly induce lipid peroxidation in membranes, resulting in viability reduction and up-regulated ROS generation [40,41]. In addition, the fucoidans isolated from *Luminaria japonica*, *Undaria pinnatifida*, *Ecklonia stolonifera,* and *Ecklonia cava* also showed antioxidant activities [13,42,43,44]. Yang et al [25] reported that fucoidan isolated from *U. pinnatifida* sporophylls with HCl solvent-assisted extraction had a protective effect against AAPH-induced oxidative stress, however, FUCS showed stronger antioxidant activity both in vitro and in vivo. FCUS also significantly suppressed the lipid peroxidation induced by AAPH. ROS includes several molecules that damage the structure of DNA and RNA, as well as oxidizes proteins and lipids to induce oxidative stress [45,46,47].

## 3. Materials and Methods

### 3.1. Material

Sporophylls of the brown algae *U. pinnatifida* were obtained from the seaweed-processing factory in Wando, South of Korea. Impurities were removed using tap water, and the samples were subjected to freeze-drying and stored at −40 °C. Trifluoroacetic acid (TFA) of analytical grade (Dae-Jung Chemicals & Metals Co., Seoul, Korea) was used for the monosaccharide compositional analysis and 97% ethanol (Dae-Jung Chemicals & Metals Co., Seoul, Korea) was used for extraction. Celluclast was purchased from Novo Nordisk (Bagsvaerd, Denmark). 3-(4,5-Dimethylthiazol-2-yl)-2,5-diphenyltetrazolium bromide (MTT), dimethyl sulfoxide (DMSO), and AAPH were purchased from Sigma-Aldrich (St. Louis, MO, USA). All other chemicals and reagents used in these experiments were of analytical grade.

### 3.2. Extraction and Purification of Fucoidan from U. pinnatifida Sporophylls

For enzyme-assisted extraction, 100 g of the dried *U. pinnatifida* sporophylls powder was mixed with 2 L of distilled water (dH_2_O) and 0.5 mL of Celluclast enzyme for 24 h in a shaking incubator (140 rpm at 50 °C). The digested material was heated at 95 °C (10 min) for enzyme inactivation. The supernatants were separated from the debris by centrifugation (3500 rpm for 20 min) following filtration. For precipitation, the hydrolysate was mixed with a 3-fold volume of ethanol (95% ethanol) and allowed to precipitate the polysaccharides overnight. After centrifugation (10,000 rpm for 15 min at 4 °C), the precipitates were dissolved in dH_2_O and subsequently treated with 4 M CaCl_2_ for 4 h to remove alginic acid interferences. The precipitate was removed in an additional centrifugation step (10,000 rpm for 15 min at 4 °C) and re-treated with three volumes of ethanol. The resulting precipitate was freeze-dried and used as the fucoidan isolated from the Celluclast enzyme extract of *U. pinnatifida* sporophylls (FCUS).

### 3.3. Characterization of FCUS

#### 3.3.1. Proximate Composition and Monosaccharide Composition

The total carbohydrate content of FCUS was analyzed by the phenol-sulfuric acid method [48] using glucose as a standard, and the protein content was analyzed by the Lowry method [49]. The sulfate content was analyzed by the BaCl_2_ gelatin method [50]. The monosaccharide composition of FCUS was analyzed according to the method described by Yang et al [25]. Briefly, FCUS was hydrolyzed using 4 M of TFA. The hydrolysate was analyzed using a Dionex ED50 Electrochemical Detector (Thermo Scientific, Waltham, MA, USA) with a CarboPac PA1 Cartridge column (4.5 mm × 50 mm, Thermo Scientific, Waltham, MA, USA). Sodium hydroxide (16 mM) was used as a mobile phase with a flow rate of 1 mL/min.

#### 3.3.2. Fourier Transform-Infrared (FT-IR) Characterization

The FCUS and commercial fucoidan (Sigma-Aldrich, St. Louis, MO, USA) were analyzed by the Potassium bromide (KBr) method using an FT-IR spectrometer (Nicole 6700, Thermo Scientific, Waltham, MA, USA), following the experimental conditions described in Fernando et al [27]. Commercial fucoidan used as a control was purchased from Sigma-Aldrich (St. Louis, MO, USA).

### 3.4. Cell culture and cell experiments

The Vero cell line (African green monkey kidney, KCKB No. 10081) was cultured in RPMI-1640 (Invitrogen-Gibco, Grand Island, NY, USA) medium containing penicillin (100 U/mL), streptomycin (100 μg/mL), and 10% FBS (Invitrogen-Gibco, Grand Island, NY, USA). The cells were maintained in an atmosphere of 5% CO_2_ at 37°C. For the measurement of reactive oxygen species (ROS) production and cell viability, Vero cells were seeded in 96-well plates at 1 × 10^5^ cells/mL for 16 h. The cells were treated with various concentrations of FCUS. After 24 h, cell viability was measured by MTT assay, as reported by Wang et al. [51], and intracellular ROS production was measured using DCF-DA assay, according to Wang et al. [51].

### 3.5. In Vivo Experiments

#### 3.5.1. Maintenance of Zebrafish

Zebrafish (*Danio rerio*) were purchased from a commercial aquarium (Jeju, Korea) and maintenance conditions were followed, as described by Kim et al [13]. Males (2) were bred with females (1) to obtain embryos, and the embryos were harvested the next morning. The harvesting method that followed was natural spawning within 30 minutes in a cell culture dish containing an embryo medium [13].

#### 3.5.2. Treatment of FCUS and AAPH to Embryos

After 8 h post fertilization, fifteen embryos were transferred to wells of a 12-well plate with embryo medium. FCUS was administered to the embryos for 1 h, and then the embryos were treated with 15 mM AAPH for 24 h. The survival rate of the embryo was observed for 7 days post-fertilization (dpf), and the heartbeat was observed at 2 dpf for 1 min using a microscope.

#### 3.5.3. Measurement of Intracellular ROS Production, Lipid Peroxidation, and Cell Death Induced by AAPH

For measurement of antioxidant activities in the zebrafish, the embryos (3 dpf) were transferred to a 12-well plate. The embryos were treated with DCFH-DA (20 μg/mL), and incubated in the dark room for 1 h at room temperature for ROS measurement. The embryos were washed using fresh medium and anesthetized using Ethyl 3-aminobenzoate methanesulfonate (Sigma-Aldrich, St. Luis, MO, USA) before observation. We placed each anesthetized larva on the slide glass, and fluorescence values were measured using a fluorescence microscope. The differences in the fluorescence intensities of the groups were noted by keeping the value of the control group fixed as the index for the comparisons. Lipid peroxidation was measured by treatment with diphenyl-1-pyrenylphosphine (DPPP, Thermo Scientific, USA). Similarly, embryos in the well plates were treated with DPPP (25 μg/mL) and incubated. Washing and anesthetizing was carried out, as mentioned above. Cell death was measured by treatment with acridine orange (Sigma-Aldrich, St. Louis, MO, USA), (7 μg/mL). The observation of zebrafish was conducted using Cool SNAP-Pro color digital camera (Olympus, Japan) according to Kim et al [13]. The fluorescence intensities of the zebrafish larvae were quantified using Image J (Version 1.51) program.

### 3.6. Statistical Analysis

All the experiments were performed in triplicate, and the data are expressed as the mean ± standard error (SE). One-way analysis of variance (ANOVA) (SPSS 11.5 statistical software) was performed to compare the mean values among treatment groups.

## 4. Conclusions

FCUS was found to be enriched in fucose and sulfate, with a prominent antioxidant effect exerted through repression of AAPH-induced ROS generation in vitro and in vivo. Thus, with further investigation into the safety and eco-friendly extraction process, FCUS has the potential to be developed as a beneficial antioxidant or nutraceutical functional ingredient, and as an alternative treatment for oxidative damage.

## Figures and Tables

**Table 1:** Yield, proximate chemical composition, and monosaccharide compositions of the celluclast enzyme extract of *U. pinnatifida* sporophylls (FCUS).

	(Dry Base, %)
Yield	6.2 ± 1.2
Carbohydrate content	66.8 ± 0.1
Protein content	2.8 ± 0.1
Sulfate content	30.4 ± 0.1
Proportion of monosaccharide	
Fucose	52.3 ± 0.92
Galactose	44.5 ± 0.16
Glucose	1.0 ± 0.01
Mannose	2.2 ± 0.02

Experiments were performed in triplicate, and the data are expressed as mean ± SE.

## References

[1] Kishi S., Uchiyama J., Baughman A.M., Goto T., Lin M.C., Tsai S.B. (2003). The zebrafish as a vertebrate model of functional aging and very gradual senescence. Exp. Gerontol..

[2] Lee S.H., Ko C.I., Jee Y., Jeong Y., Kim M., Kim J.S., Jeon Y.J. (2013). Anti-inflammatory effect of fucoidan extracted from Ecklonia cava in zebrafish model. Carbohydr. Polym..

[3] Heo S.-J., Park E.-J., Lee K.-W., Jeon Y.-J. (2005). Antioxidant activities of enzymatic extracts from brown seaweeds. Bioresource Technol..

[4] Merino S.T., Cherry J. (2007). Progress and challenges in enzyme development for biomass utilization. Biofuels.

[5] Synytsya A., Kim W.J., Kim S.M., Pohl R., Synytsya A., Kvasnicka F., Copikova J., Park Y.I. (2010). Structure and antitumour activity of fucoidan isolated from sporophyll of Korean brown seaweed Undaria pinnatifida. Carbohydr. Polym..

[6] Koo J.-G., Choi Y.-S., Kwak J.-K. (2001). Blood-anticoagulant activity of fucoidans from sporophylls of Undaria pinnatifida, Laminaria religiosa, Hizikia fusiforme and Sargassum fulvellum in Korea. Korean J. Fish. Aquat. Sci..

[7] Jiang Z.D., Okimura T., Yokose T., Yamasaki Y., Yamaguchi K., Oda T. (2010). Effects of sulfated fucan, ascophyllan, from the brown Alga Ascophyllum nodosum on various cell lines: A comparative study on ascophyllan and fucoidan. J. Biosci. Bioeng..

[8] Li B., Lu F., Wei X., Zhao R. (2008). Fucoidan: Structure and bioactivity. Molecules.

[9] Lim S.J., Aida W.M.W., Maskat M.Y., Mamot S., Ropien J., Mohd D.M. (2014). Isolation and antioxidant capacity of fucoidan from selected Malaysian seaweeds. Food Hydrocolloid..

[10] Boo H.J., Hyun J.H., Kim S.C., Kang J.I., Kim M.K., Kim S.Y., Cho H., Yoo E.S., Kang H.K. (2011). Fucoidan from Undaria pinnatifida induces apoptosis in A549 human lung carcinoma cells. Phytother. Res..

[11] Kim M.J., Jeon J., Lee J.S. (2014). Fucoidan prevents high-fat diet-induced obesity in animals by suppression of fat accumulation. Phytother. Res..

[12] Mak W., Hamid N., Liu T., Lu J., White W.L. (2013). Fucoidan from New Zealand Undaria pinnatifida: Monthly variations and determination of antioxidant activities. Carbohydr. Polym..

[13] Kim E.A., Lee S.H., Ko C.I., Cha S.H., Kang M.C., Kang S.M., Ko S.C., Lee W.W., Ko J.Y., Lee J.H. (2014). Protective effect of fucoidan against AAPH-induced oxidative stress in zebrafish model. Carbohydr. Polym..

[14] Lee J.B., Hayashi K., Hashimoto M., Nakano T., Hayashi T. (2004). Novel antiviral fucoidan from sporophyll of Undaria pinnatifida (Mekabu). Chem. Pharm. Bull. (Tokyo).

[15] Puri M., Sharma D., Barrow C.J. (2012). Enzyme-assisted extraction of bioactives from plants. Trends Biotechnol..

[16] Teo C.C., Tan S.N., Yong J.W., Hew C.S., Ong E.S. (2010). Pressurized hot water extraction (PHWE). J. Chromatogr. A.

[17] Sanjeewa K.K.A., Lee J.S., Kim W.S., Jeon Y.J. (2017). The potential of brown-algae polysaccharides for the development of anticancer agents: An update on anticancer effects reported for fucoidan and laminaran. Carbohydr. Polym..

[18] Maruyama H., Tamauchi H., Hashimoto M., Nakano T. (2003). Antitumor activity and immune response of Mekabu fucoidan extracted from Sporophyll of Undaria pinnatifida. In Vivo.

[19] Wijesinghe W.A.J.P., Jeon Y.J. (2012). Biological activities and potential industrial applications of fucose rich sulfated polysaccharides and fucoidans isolated from brown seaweeds: A review. Carbohydr. Polym..

[20] Maier T., Schieber A., Kammerer D.R., Carle R. (2009). Residues of grape (Vitis vinifera L.) seed oil production as a valuable source of phenolic antioxidants. Food Chem..

[21] Kang M.C., Kim S.Y., Kim Y.T., Kim E.A., Lee S.H., Ko S.C., Wijesinghe W.A., Samarakoon K.W., Kim Y.S., Cho J.H. (2014). In vitro and in vivo antioxidant activities of polysaccharide purified from aloe vera (Aloe barbadensis) gel. Carbohydr. Polym..

[22] Ko S.C., Kang N., Kim E.A., Kang M.C., Lee S.H., Kang S.M., Lee J.B., Jeon B.T., Kim S.K., Park S.J. (2012). A novel angiotensin I-converting enzyme (ACE) inhibitory peptide from a marine Chlorella ellipsoidea and its antihypertensive effect in spontaneously hypertensive rats. Process Biochem..

[23] Heo S.J., Park P.J., Park E.J., Kim S.K., Jeon Y.J. (2005). Antioxidant activity of enzymatic extracts from a brown seaweed Ecklonia cava by electron spin resonance spectrometry and comet assay. Eur. Food Res. Technol..

[24] Nishino T., Nishioka C., Ura H., Nagumo T. (1994). Isolation and partial characterization of a noval amino sugar-containing fucan sulfate from commercial Fucus vesiculosus fucoidan. Carbohydr. Res..

[25] Yang H.W., Oh J.Y., Kim E.A., Kim S.Y., Kim J.I., Jeon Y.J. (2015). Antioxidant Effect of Fucoidan from Miyeokgui, Marine Alga in Zebrafish Model. Journal of chitin and chitosan.

[26] Patankar M.S., Oehninger S., Barnett T., Williams R.L., Clark G.F. (1993). A Revised Structure for Fucoidan May Explain Some of Its Biological-Activities. J. Biol. Chem..

[27] Fernando I.P.S., Sanjeewa K.K.A., Samarakoon K.W., Lee W.W., Kim H.S., Kang N., Ranasinghe P., Lee H.S., Jeon Y.J. (2017). A fucoidan fraction purified from Chnoospora minima; a potential inhibitor of LPS-induced inflammatory responses. Int. J. Biol. Macromol..

[28] Fernando I.P.S., Sanjeewa K.K.A., Samarakoon K.W., Lee W.W., Kim H.S., Kim E.A., Gunasekara U.K.D.S.S., Abeytunga D.T.U., Nanayakkara C., De Silva E.D. (2017). FTIR characterization and antioxidant activity of water soluble crude polysaccharides of Sri Lankan marine algae. Algae-Seoul.

[29] Kang N., Kim S.-Y., Rho S., Ko J.-Y., Jeon Y.-J. (2017). Anti-fatigue activity of a mixture of seahorse (Hippocampus abdominalis) hydrolysate and red ginseng. Fish. Aquat. Sci..

[30] Kim H.-H., Kim H.-S., Ko J.-Y., Kim C.-Y., Lee J.-H., Jeon Y.-J. (2016). A single-step isolation of useful antioxidant compounds from Ishige okamurae by using centrifugal partition chromatography. Fish. Aquat. Sci..

[31] Wang L., Jo M.J., Katagiri R., Harata K., Ohta M., Ogawa A., Kamegai M., Ishida Y., Tanoue S., Kimura S. (2018). Antioxidant effects of citrus pomace extracts processed by super-heated steam. Lwt-Food Sci. Technol..

[32] Fernando I.P., Kim M., Son K.T., Jeong Y., Jeon Y.J. (2016). Antioxidant Activity of Marine Algal Polyphenolic Compounds: A Mechanistic Approach. J. Med. Food.

[33] Kim Y.S., Hwang J.W., Sung S.H., Jeon Y.J., Jeong J.H., Jeon B.T., Moon S.H., Park P.J. (2015). Antioxidant activity and protective effect of extract of Celosia cristata L. flower on tert-butyl hydroperoxide-induced oxidative hepatotoxicity. Food Chem..

[34] Qi H.M., Zhao T.T., Zhang Q.B., Li Z., Zhao Z.Q., Xing R. (2005). Antioxidant activity of different molecular weight sulfated polysaccharides from Ulva pertusa Kjellm (Chlorophyta). J. Appl. Phycol..

[35] Costa L.S., Fidelis G.P., Cordeiro S.L., Oliveira R.M., Sabry D.A., Camara R.B., Nobre L.T., Costa M.S., Almeida-Lima J., Farias E.H. (2010). Biological activities of sulfated polysaccharides from tropical seaweeds. Biomed. Pharmacother..

[36] Mugoni V., Camporeale A., Santoro M.M. (2014). Analysis of oxidative stress in zebrafish embryos. J. Vis. Exp..

[37] Kang M.C., Cha S.H., Wijesinghe W.A., Kang S.M., Lee S.H., Kim E.A., Song C.B., Jeon Y.J. (2013). Protective effect of marine algae phlorotannins against AAPH-induced oxidative stress in zebrafish embryo. Food Chem..

[38] Cho S.H., Heo S.J., Yang H.W., Ko E.Y., Jung M.S., Cha S.H., Ahn G., Jeon Y.J., Kim K.N. (2019). Protective Effect of 3-Bromo-4,5-Dihydroxybenzaldehyde from Polysiphonia morrowii Harvey against Hydrogen Peroxide-Induced Oxidative Stress In Vitro and In Vivo. J. Microbiol. Biotechnol..

[39] Lee S.H., Ko C.I., Ahn G., You S., Kim J.S., Heu M.S., Kim J., Jee Y., Jeon Y.J. (2012). Molecular characteristics and anti-inflammatory activity of the fucoidan extracted from Ecklonia cava. Carbohydr. Polym..

[40] Park J.E., Yang J.H., Yoon S.J., Lee J.H., Yang E.S., Park J.W. (2002). Lipid peroxidation-mediated cytotoxicity and DNA damage in U937 cells. Biochimie.

[41] Socrier L., Rosselin M., Giraldo A.M.G., Chantemargue B., Di Meo F., Trouillas P., Durand G., Morandat S. (2019). A Novel Nitrone-Trolox Conjugate Inhibits Membrane Lipid Oxidation Through Synergistic Antioxidant Effects. Biophys. J..

[42] Wang J., Zhang Q., Zhang Z., Song H., Li P. (2010). Potential antioxidant and anticoagulant capacity of low molecular weight fucoidan fractions extracted from Laminaria japonica. Int. J. Biol. Macromol..

[43] Li L.H., Xue C.H., Xue Y., Li Z.J., Fu X.Y. (2006). The effects of fucoidans from Laminaria japonica on AAPH mediated oxidation of human low-density lipoprotein. Acta Oceanol. Sin..

[44] Kuda T., Kunii T., Goto H., Suzuki T., Yano T. (2007). Varieties of antioxidant and antibacterial properties of Ecklonia stolonifera and Ecklonia kurome products harvested and processed in the Noto Peninsula, Japan. Food Chem..

[45] Wu D., Yotnda P. (2011). Production and detection of reactive oxygen species (ROS) in cancers. J. Vis. Exp..

[46] Papaharalambus C.A., Griendling K.K. (2007). Basic mechanisms of oxidative stress and reactive oxygen species in cardiovascular injury. Trends Cardiovasc. Med..

[47] Blagosklonny M.V. (2008). Aging: ROS or TOR. Cell Cycle.

[48] Dubois M., Gilles K.A., Hamilton J.K., Rebers P., Smith F. (1956). Colorimetric method for determination of sugars and related substances. Anal. Chem..

[49] Lowry O.H., Rosebrough N.J., Farr A.L., Randall R.J. (1951). Protein measurement with the Folin phenol reagent. J. Biol. Chem..

[50] Dodgson K.S., Price R.G. (1962). A note on the determination of the ester sulphate content of sulphated polysaccharides. Biochem. J..

[51] Wang L., Ryu B., Kim W.S., Kim G.H., Jeon Y.J. (2017). Protective effect of gallic acid derivatives from the freshwater green alga Spirogyra sp against ultraviolet B-induced apoptosis through reactive oxygen species clearance in human keratinocytes and zebrafish. Algae.

